# APC Moonlights to Prevent Wnt Signalosome Assembly

**DOI:** 10.1016/j.devcel.2018.02.018

**Published:** 2018-03-12

**Authors:** Ian John McGough, Jean-Paul Vincent

**Affiliations:** 1 The Francis Crick Institute, 1 Midland Road, London NW1 1AT, UK

## Abstract

The scaffold protein APC has a well-known function in ensuring β-catenin destruction. In this issue of *Developmental Cell*, Saito-Diaz et al. (2018) uncover another role for APC in Wnt signaling: to prevent clathrin-dependent signalosome formation in the absence of ligand.

## Main Text

**Figure 1:**
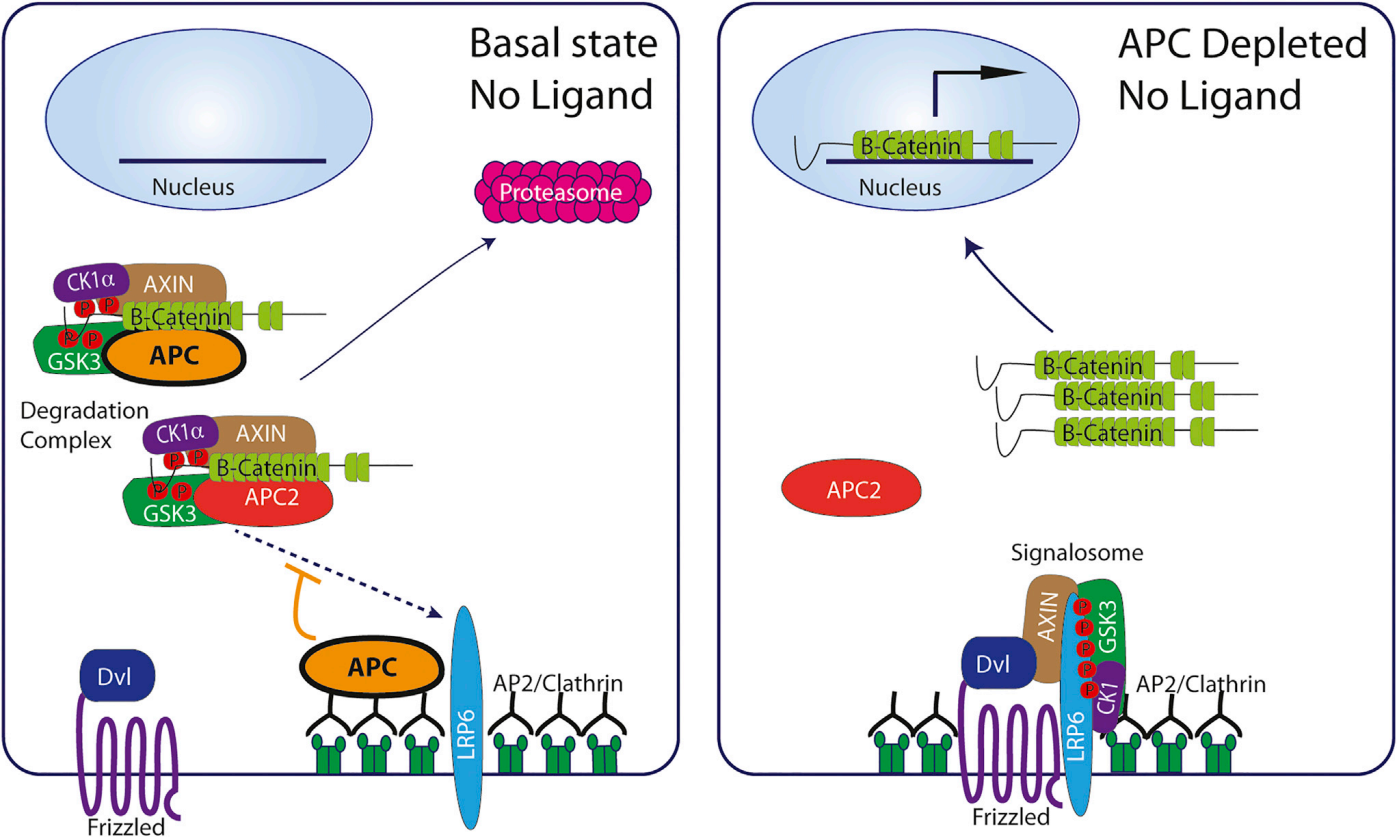
APC’s Two Means of Suppressing Wnt Signaling in the Absence of Ligand APC and APC2 are redundant in the destruction complex, which phosphorylates and targets β-catenin for degradation. Saito-Diaz et al. (2018) show that APC, but not APC2, has an additional function in preventing signalosome formation in the absence of Wnt ligand. In APC-depleted cells, APC2 cannot compensate for APC’s role in preventing clathrin-dependent signalosome formation. This leaves APC2 without a destruction complex to participate in. Thus, in the absence of APC, the signalosome forms, LRP6 is phosphorylated, and β-catenin is activated, along with downstream transcription.

Wnts are secreted proteins that control many aspects of development and adult homeostasis through modulating the activity of β-catenin. Insufficient Wnt-β-catenin signaling leads to developmental defects and loss of stem cells, whereas excess signaling has been abundantly linked to tumorigenesis. In the absence of Wnt ligand, the so-called destruction complex—which comprises casein kinase 1α, glycogen synthase kinase 3 (GSK3), and two scaffold proteins, Axin and APC—ensures that β-catenin is continuously targeted for proteasomal degradation through phosphorylation by GSK3 and subsequent ubiquitylation (MacDonald and He, 2012). In the presence of Wnt, the receptors Frizzled (Fz) and LRP5/6 recruit Dishevelled (DVL), Axin, and GSK3 to seed the formation of a “signalosome” that disrupts the destruction complex, allowing β-catenin nuclear translocation and activation of the Wnt transcriptional program (MacDonald and He, 2012). This form of control by negative regulation is susceptible to mutations that cause unbridled signal transduction with detrimental effects. Indeed, mutations in APC cause excess Wnt-β-catenin signaling and are a major cause of colon cancer. Diseases caused by these mutations are seen as particularly problematic because they are not expected to be sensitive to therapies that target upstream components of the signal transduction machinery. However, as described by Saito-Diaz et al. (2018) in this issue of *Developmental Cell*, activation of β-catenin signaling by loss of APC still responds to manipulation of upstream components. In a series of elegant experiments, Saito-Diaz et al. demonstrate that loss of APC leads to spontaneous signalosome formation without Wnt ligand in a manner that requires clathrin, DVL, and the receptor complex (Figure 1).

The authors first show that inhibition of LRP6 in colorectal-cancer cell lines (CRCs) carrying mutations in APC, or in cells lacking APC by CRISPR-mediated knockout, reverses β-catenin signaling. They further validate this requirement for LRP6 with a blocking antibody that they developed against a central region of the extracellular region of this receptor. Perturbation of other signalosome components, Fz or DVL, also prevented pathway activation in APC mutant cells, indicating that they, too, are required for β-catenin signaling in APC mutant cells. Moreover, LRP6 was found in a dense fraction indicative of signalosome in lysates prepared from APC mutant cells. Therefore, it appears that, in APC-deficient cells, the signalosome forms and depletion of signalosome components allows the destruction complex to recover its function, most likely through the activity of APC2, a paralog of APC (Daly et al., 2017). These events do not seem to require the ligand because inhibition of Porcupine, an enzyme that adds a signaling-critical lipid to all Wnts (Zhai et al., 2004), had no impact on the activation of downstream signaling in APC mutant cells.

To confirm their findings in a physiological context, the authors turned to *ex vivo* organoid cultures derived from intestinal adenomas of APC^min^ mice and to the *Drosophila* adult midgut. As expected, LRP6 suppression inhibited the growth of APC^min^ organoids. In the *Drosophila* adult midgut, as in the adult mammalian intestine, loss of APC aberrantly activates the Wnt pathway, leading to increased expression of Wnt target genes, overproliferation of intestinal stem cells, and disruption of epithelial polarity. All three phenotypes were reversed by genetic disruption of signalosome components, but not by mutations that target the ligand (Wingless) or its secretion, mirroring the findings from cell culture. How does signalosome assembly induced by loss of APC occur in the absence of ligand? As the authors show, this involves an APC-modulated, clathrin-dependent mechanism.

Clathrin, a key effector of receptor-mediated endocytosis, is essential for Wnt signal transduction, although the underlying mechanism is still subject to debate. There is broad agreement that DVL promotes the recruitment of clathrin at ligand-receptor complexes. Specifically, DVL directly interacts with—and stimulates—phosphatidylinositol 4-phosphate 5-kinase 1 (PIP5K1) (Pan et al., 2008), leading to a local increase in phosphatidylinositol 4,5-bisphosphate (PI4,5P_2_), which in turn recruits clathrin and its adaptor AP2. AP2 and clathrin are then further enriched at these domains through the AP2-binding motifs present in LRP6 and DVL (Kim et al., 2013). One model suggests that such recruitment leads to endocytosis and subsequent sequestration of GSK3 inside intraluminal vesicles (ILVs) of late endosomes, preventing it from phosphorylating β-catenin left behind in the cytoplasm (Taelman et al., 2010). An alternative model, currently receiving wider support, is that the accumulation of DVL at the receptor complex leads to the assembly of a high-avidity platform that attracts Axin and GSK3, allowing GSK3 to phosphorylate PPPSPXS/T motifs in the cytoplasmic tail of LRP6 (MacDonald and He, 2012). These phosphorylated motifs of LRP6 would then act as competitive inhibitors of GSK3, preventing phosphorylation of β-catenin (Stamos et al., 2014).

Clathrin is known to be required for ligand-induced Wnt signaling but it will come as a surprise to many that, as shown by Saito-Diaz et al. (2018), inhibition of Dynamin, a mediator of endocytosis (including clathrin-mediated endocytosis), suppressed the accumulation of β-catenin caused by APC depletion. Equally surprising is the finding that treatment with the new anti-LRP6 blocking antibody prevented the internalization of LRP6 seen upon APC depletion. These findings led the authors to consider the possibility that APC could affect signalosome formation and hence inactivation of the destruction complex, while at the same time being an essential component of the destruction complex. Together, these activities would lock signal transduction in the OFF state, as long as no ligand is present. Consistent with a possible role at the signalosome, the authors showed that APC physically interacts with AP2 and clathrin. They suggest, therefore, that APC, through its binding to clathrin, moonlights as a gatekeeper that blocks signalosome formation, preventing spurious activation in the absence of ligand. The generation of point mutations that prevent APC from binding to AP2/clathrin would help to confirm whether this interaction is indeed required for gatekeeping activity. One implication of this study is that clathrin must switch from inhibiting signalosome formation in the absence of Wnt to promoting it in the presence of Wnt. Further work is needed to uncover the underlying molecular mechanism.

The results of Saito-Diaz et al. (2018) explain why APC mutations are oncogenic despite the fact that APC2 can take part in the destruction complex. This is because APC2 does not inhibit signalosome formation and is therefore unable to complement both activities of APC. In mice, APC2 might fully complement APC, because deletion of either APC or APC2 alone has no impact on Wnt signaling, at least in the mammary epithelium (Daly et al., 2017). One important implication of the present work is that restoring gatekeeper activity by independently preventing signalosome formation could help bring down β-catenin levels in APC-deficient cells. Accordingly, cancers caused by mutations in APC could conceivably be treated with small-molecule inhibitors or antibodies that prevent signalosome formation, such as the anti-LRP6 antibody used in this study. Although this approach brings fresh hope, it is not ready for implementation because global inhibition of Wnt signaling, e.g., with anti-LRP6, is not desirable. A more targeted strategy will be needed to allow normal ligand-induced signalosome formation while preventing ligand-independent signalosome formation in APC mutant cells. A more detailed understanding of the interactions between APC and the endocytic machinery in future work will allow such an approach to progress beyond the drawing board.
